# An investigation of eye lens dose for gamma knife treatments of trigeminal neuralgia

**DOI:** 10.1120/jacmp.v1i4.2632

**Published:** 2000-09-01

**Authors:** Lijun Ma, Lawrence Chin, Mehrdad Sarfaraz, David Shepard, Cedric Yu

**Affiliations:** ^1^ Department of Radiation Oncology University of Maryland School of Medicine Baltimore MD 21201; ^2^ Department of Neurosurgery University of Maryland School of Medicine Baltimore MD 21201

**Keywords:** gamma knife, stereotactic radiosurgery, trigeminal neuralgia, dosimetry

## Abstract

Stereotactic Gamma Knife radiosurgery has been widely used for treating trigeminal neuralgia (TN). A single large fractional dose of 7000 to 9000 cGy is commonly prescribed as the maximum dose for these treatments. For this reason, if a small percentage of the prescribed dose such as 2–3 % scattered to the eye, it could reach or even exceed the tolerance dose of the lens. For several TN cases, we found that the Leksell Gamma Plan system calculates the lens dose about 0.5–2 % of the maximum dose independent of the use of eye shielding. These dose values are significantly high and it motivated us to investigate the lens dose for the TN patients treated with stereotactic Gamma Knife radiosurgery. Phantom studies and *in vivo* dosimetry measurements were carried out for six patients treated at our institution. The average dose to the lens ipsilateral to the treated nerve was measured to be 7.7±0.6 cGy. Based on the biological model of Lyman and Emami [Int. J. Radiat. Oncol. Biol. Phys. **21,** 109–122 (1991)], the probability of the lens complication (cataract) was determined to be 0.1%. Our findings suggest that few TN patients would develop cataracts after receiving Gamma Knife radiosurgery.

PACS number(s): 87.53.–j, 87.66.–a

## I. INTRODUCTION

Stereotactic Gamma Knife radiosurgery is considered to be one of the safest and most effective treatment modality for trigeminal neuralgia (TN).^1^ ^–^ ^3^ Since the procedure reimbursement has been accepted by Medicare, there has been a dramatic increase in the number of patients receiving Gamma Knife radiosurgery for trigeminal neuralgia. According to a recent survey by the Leksell Gamma Knife Society, trigeminal neuralgia is one of the most commonly treated indications for Gamma Knife procedures in the United States.

To carry out an effective TN procedure, it is common that a single large fractional dose of 7000 to 9000 cGy is used to irradiate the affected nerve.^1^ Because of the large delivered dose, a scatter dose even 2% of the total dose could reach or even exceed the tolerance dose of the eye lens^5^ (about 150 cGy). Based on the predictions of the Gamma Knife treatment planning system, we found that the dose to the eye lens sometimes exceeds 100 cGy. This value was significantly higher than expected and it motivated us to carry out clinical physics studies to investigate the lens dose for trigeminal neuralgia patients treated with stereotactic Gamma Knife radiosurgery.

## II. MATERIALS AND METHODS

**Figure 1:**
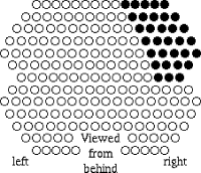
A special collimator shielding pattern used for treating trigeminal neuralgia patients at our institution.

**Figure 2:**
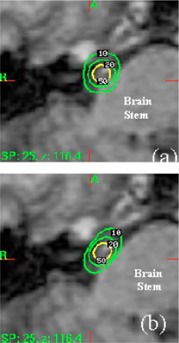
(Color) Effects of the shielding pattern in on reducing the dose to the brain stem. (a) Dose distribution before the pattern is applied. (b) Dose distribution after the pattern is applied. Note that lower isodose lines as become tangential to the edge of the brain stem after the shielding being applied.

Phantom studies and *in vivo* dosimetry measurements on six patients were carried out to measure the dose to the eye lens. All patients were treated at the University of Maryland Gamma Knife Center. Four of the patients were treated in the supine position with proper collimator eye shields and two of the patients were treated in the prone position without collimator eye shields. The eyeshields were generated by swapping the blank collimators with the 4‐mm collimators that directly irradiate the eye. The location and the number of the plugged collimators were determined for each treatment shot by shining a flashlight beam with patient locking in the treatment position. Additionally, a special plugging pattern was used for all patients. The plugging pattern is shown in Fig. 1. This plugging pattern was designed to reduce the dose to the brain stem. The dosimetry effects of the plugging pattern are illustrated in Fig. 2.

**Figure 3:**
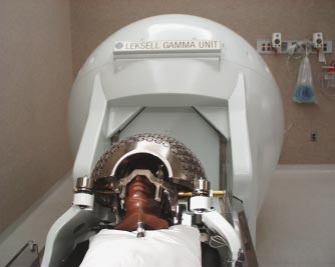
(Color) Experimental setup for the phantom studies.

High sensitivity thermoluminescence dosimeter (TLD) chips (3 mm×3 mm×0.5 mm) were used for the *in vivo* dosimetry measurements. Three to four TLD chips were placed on the eyelids of the patients while their eyes were closed during the treatments. The average distance between the TLD chips and the isodose center was approximately 7.5 cm. This was determined by using the line measurement tool from the Leksell Gamma Plan (LGP) system for each patient. Additionally, phantom studies were conducted with the TLD chips using an anthropomorphic head phantom (Humanoid Systems, Hawthorne, CA). The experimental setup is shown in Fig. 3. The effects of the collimator shielding on the lens dose were evaluated using separate measurements. For all patients and phantom studies, 7500 cGy was prescribed to the maximum dose, i.e., 100% isodose line and delivered with a single shot using the 4‐mm helmet.

## III. RESULTS

**Figure 4:**
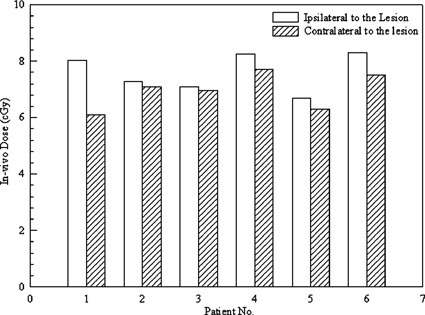
Results of the *in vivo* dosimetry measurements for the TN patients.

The results for the *in vivo* dosimetry measurements are summarized in Fig. 4. On average, the scatter dose was measured to be 7.6±0.6 cGy for the eye lens ipsilateral to the lesion, and 6.9±0.6 cGy for the eye lens contralateral to the lesion. There was no observable difference for patients treated either in the supine or the prone positions. The average lens dose for the phantom studies was measured to be 8.1±0.7 cGy. The eye shield collimators were found to have no significant effects on the measured lens dose. The lowest lens dose was measured to be 6.9±0.6 cGy cGy with the eye shields in place for the phantom while the highest dose was measured to be 9.1±0.7 cGy cGy without the eye shields for the phantom.

Using the error‐weighted average value of 7.7±0.6 cGy for all the patients, the complication probability for the eye lens (cataract requiring intervention) was determined to be 0.1%. This value was calculated based on the Lyman‐Emami biological model and the published data for eye lens irradiation.^3^ ^,^ ^4^ To fix the empirical parameters for the calculation, we used the following single fractional tolerance dose for the whole lens irradiation: $D_{50}=8\;\text{Gy},\;D_{5}=2\;\text{Gy}$.

## IV. DISCUSSION

The measured eye dose is significantly lower than the value predicted by the Leksell Gamma Knife treatment planning system (version 5.20). This could be caused by the rounding approximations in the dose models. The dose calculation needs to be extremely accurate (within 0.01%) for each source calculation in order to predict the 1–2% dose variation for a total of 201 independent source irradiations for a Gamma Knife unit (model *U*). Because of this, we recommend that Gamma Knife users use necessary caution when planning and evaluating a dose point that is relatively far from the isocenter. For this study, our calculated complication rate of 0.1% suggests that few TN patients would develop cataracts after receiving Gamma Knife radiosurgery.

## ACKNOWLEDGMENTS

The authors would like to thank Terri Biggins for assistance.
